# Supplementary figures and images for: Co-chaperone involvement in knob biogenesis implicates host-derived chaperones in malaria virulence
Source: PLoS Pathog. 2021 Oct 6;17(10):e1009969. doi: 10.1371/journal.ppat.1009969 (PMC8544838; doi:10.1371/journal.ppat.1009969)

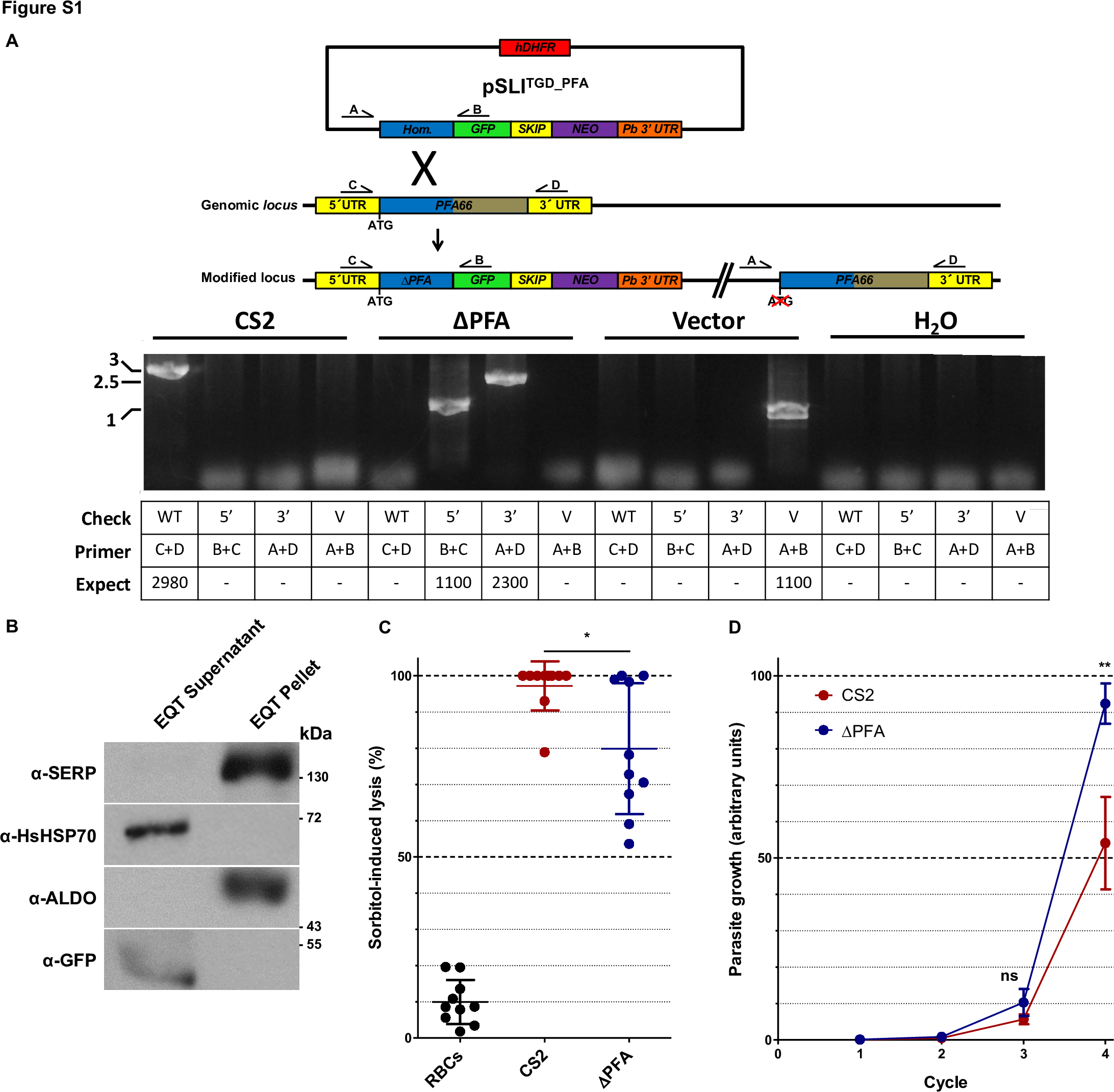

Supplement: S1 Fig — A) Upper panel, schematic of integration PCR strategy; Lower, integration PCR in original uncropped version. Table shows primer combinations and predicted result. B) An equinatoxin lysis experiment demonstrates export of truncated PFA::GFP. Equinatoxin (EQT) treatment selectively lyses the RBC membrane but leaves the PVM and PPM intact. Consequentially, parasite proteins exported to the host cell are found in the supernatant, while other parasite proteins remain in the pellet. Detection of the PV protein SERP and the parasite protein ALDO in the pellet fraction demonstrates intactness of the PV membrane and PPM, respectively. Truncated PFA::GFP was detected alongside human HSP70 in the supernatant fraction, demonstrating its export to the iRBC. C) ΔPFA display a slight decrease in NPP activity when compared to CS2. iRBCs were incubated with the hypotonic agent sorbitol, and NPP activity was assessed by measuring the OD of the supernatant. Results are shown for ten biological replicates. D) Growth of CS2 and ΔPFA was measured over four cycles via flow cytometry of DAPI-stained, fixed parasites. ΔPFA show a slight growth advantage over CS2 in the last cycle. Results are shown for three independent experiments. (TIF) [file ppat.1009969.s001.tif]

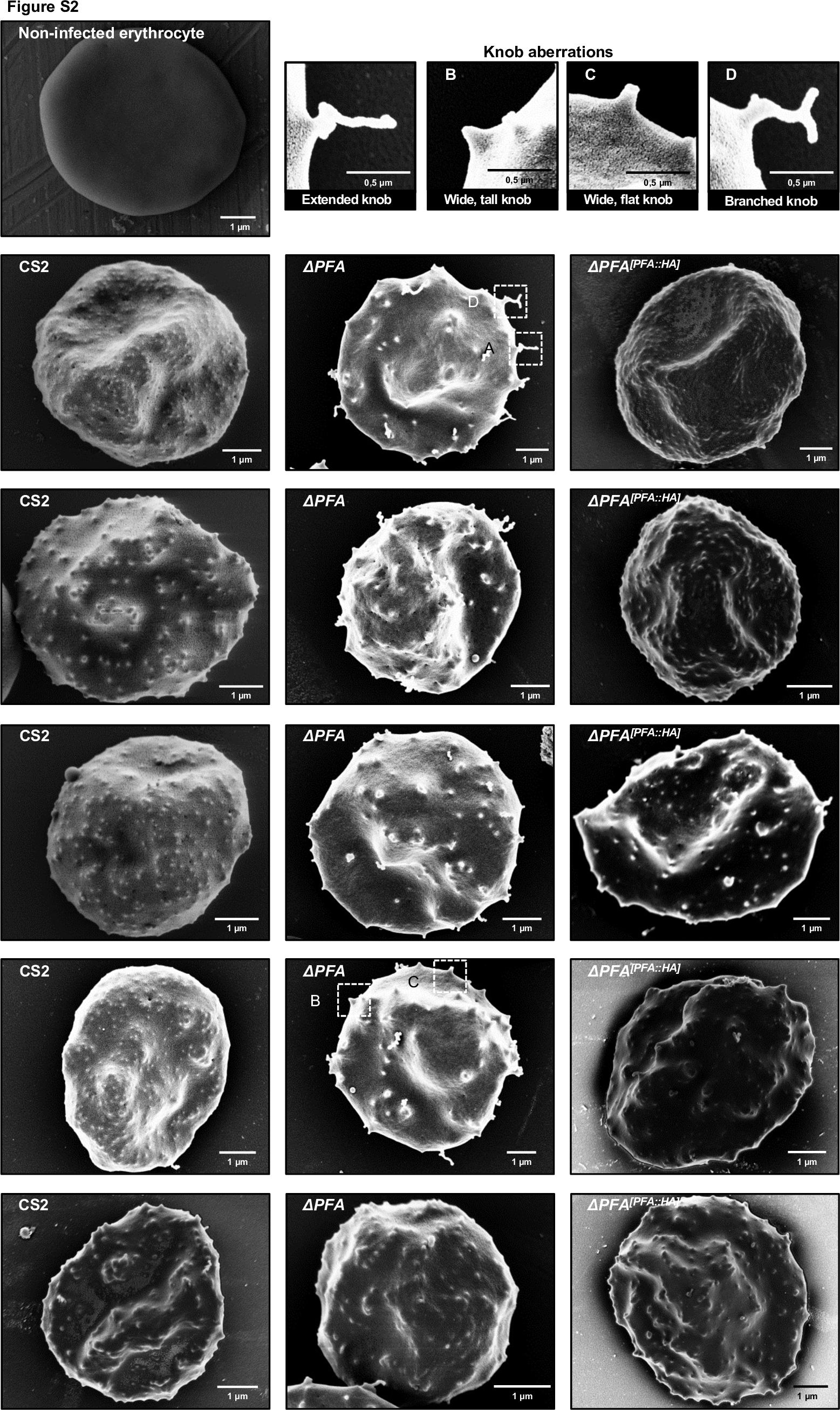

Supplement: S2 Fig — SEM image of a non-iRBC and additional SEM images of CS2, ΔPFA and ΔPFA[PFA::HA]. Included is a display of aberrant eKnob morphologies. (TIF) [file ppat.1009969.s002.tif]

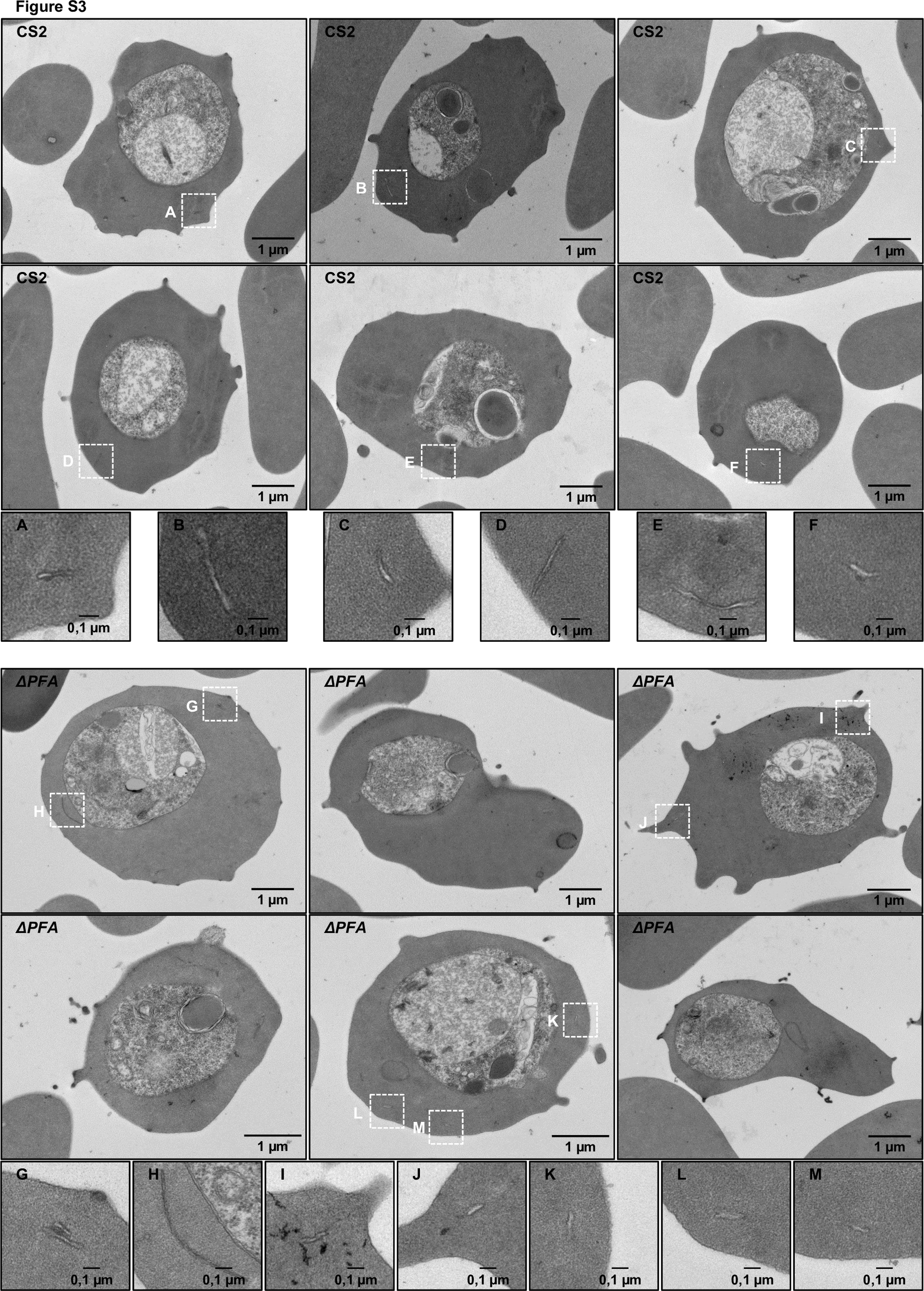

Supplement: S3 Fig — Marked areas are enlarged in the bottom panels to show Maurer´s cleft morphology in more detail. (TIF) [file ppat.1009969.s003.tif]

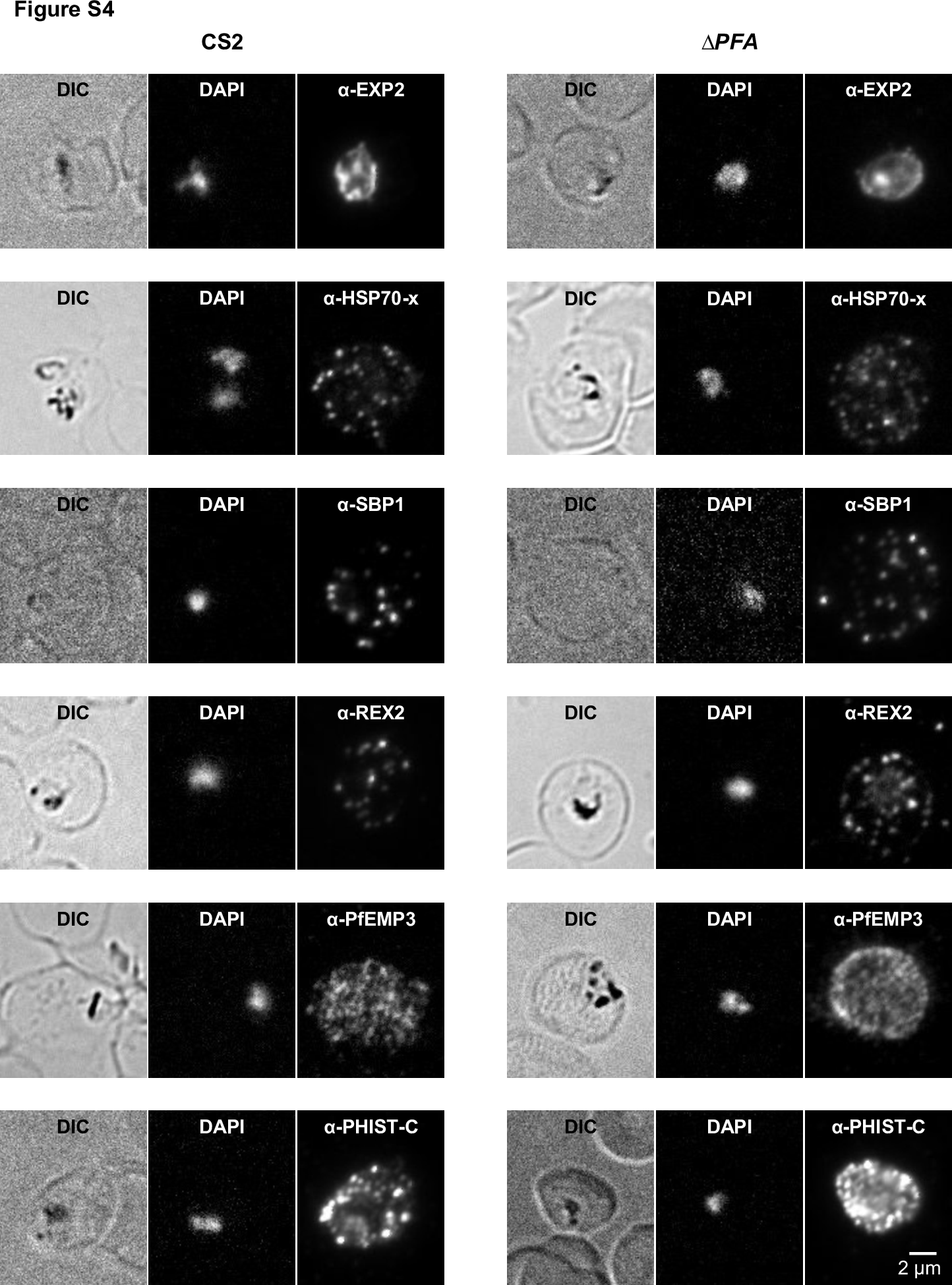

Supplement: S4 Fig — Investigation of marker protein localisation using specific antisera in MeOH acetone-fixed ΔPFA with an IFA assay. No drastic difference in the localisation of EXP2, HSP70x, SBP1, REX2, PFEMP3, or PHISTC was found. (TIF) [file ppat.1009969.s004.tif]

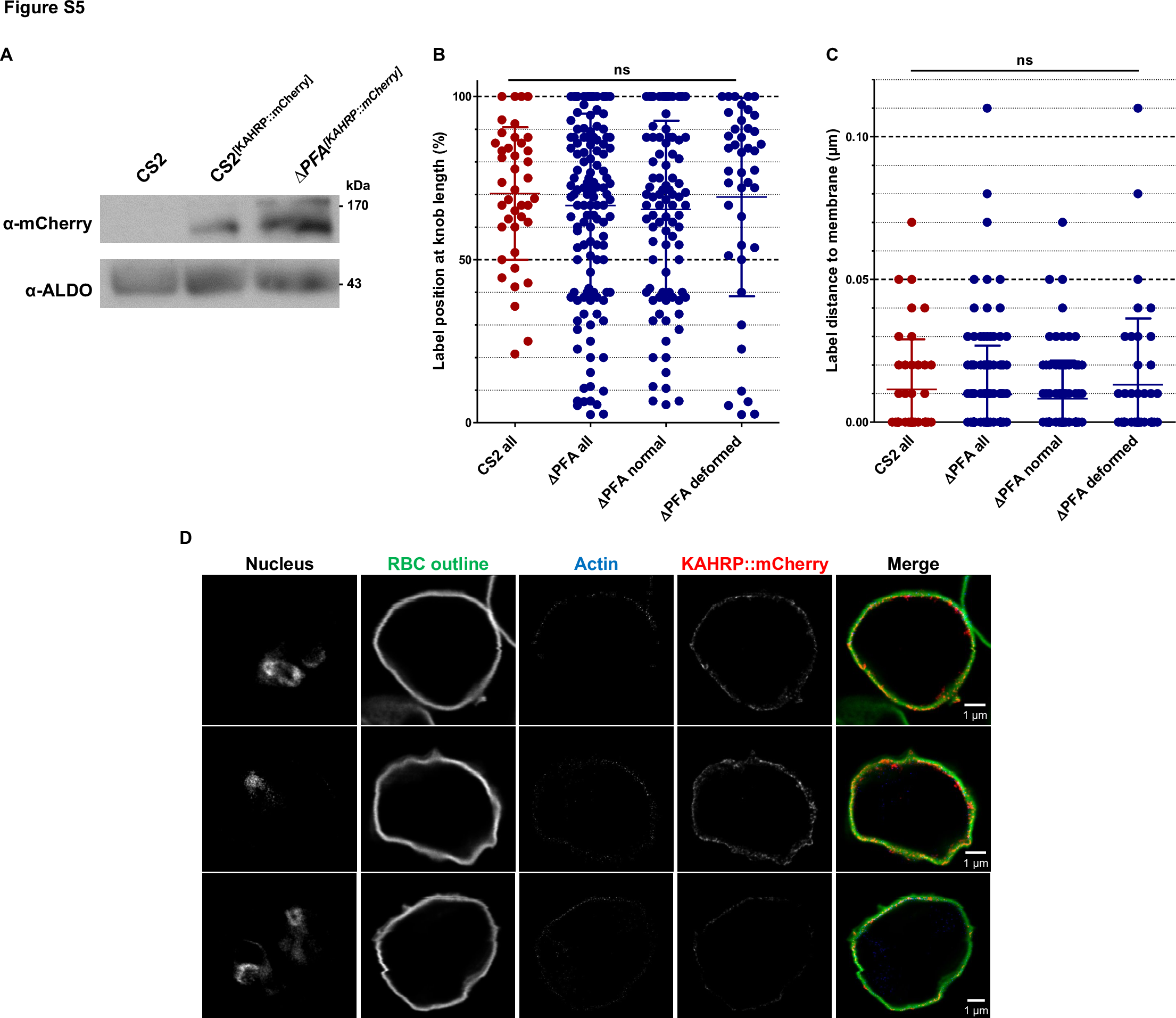

Supplement: S5 Fig — A) Verification of CS2[KAHRP::mCherry] and ΔPFA[KAHRP::mCherry] via Western blot verifies the production of KAHRP::mCherry protein in the cell lines using an α-mCherry antibody (expected MW 92kDa). The parasite protein aldolase (ALDO) was used as a loading control. B) Investigation of label distribution in the α-KAHRP immuno-TEM. Distance of label from the base of the knob was measured using ImageJ and expressed relative to the length of the entire knob in percentages (0% being the base and 100% the top). The distribution of label along the full length of the knobs did not differ between the strains and knob types, C) Distance of label to the closest membrane was measured using ImageJ, revealing no difference between the strains and knob types. D) RSTED images of CS2[KAHRP::mCherry] reveal close association of KAHRP::mCherry with the cytoskeleton and glycocalyx. Larger aggregates of KAHRP::mCherry were, in contrast to ΔPFA[KAHRP::mCherry], not observed. (TIF) [file ppat.1009969.s005.tif]

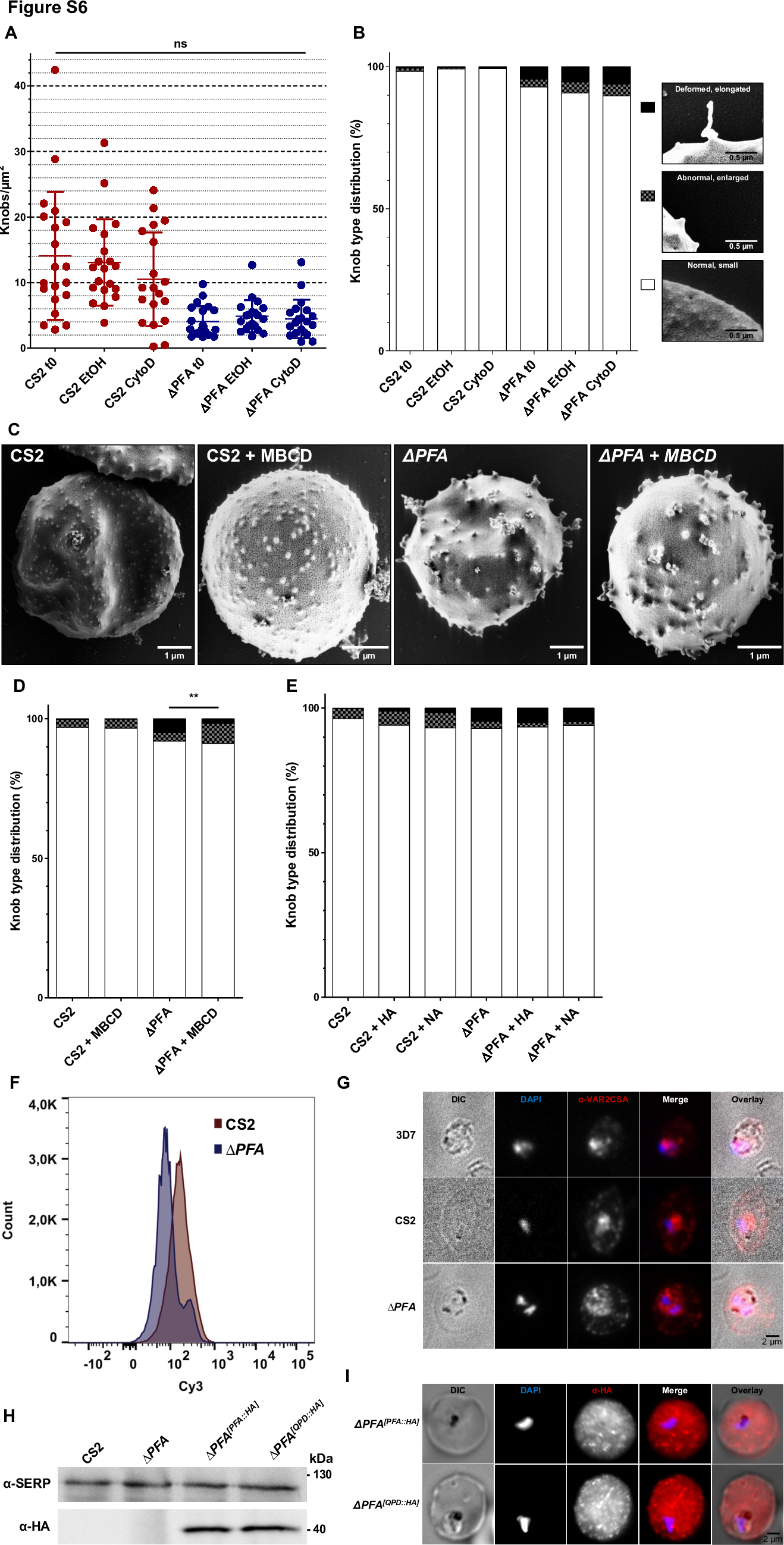

Supplement: S6 Fig — A) Treatment with the actin depolymerizing agent cytochalasin-D does not resolve knob density (A) and eKnob morphology (B, N = 15). Investigation of treatment with the lipid raft disruptor MBCD (C, D) the glucosidases hyaluronidase (HA) and neuraminidase (NA) (E) on knob-type distribution in the two cell lines. N = 15. F) Concatenation of all iRBC data from the experiments in Fig 5B also shows a decrease in surface exposed in ΔPFA across all experiments. Total number of single cells: 1,798,268 (CS2) and 1,799,274 (ΔPFA). G) Investigation whether MeOH-fixed parasites with an α-VAR2CSA antibody demonstrate that both CS2 and ΔPFA express var2CSA to similar levels. H) Comparison of episomally expressed trans-gene (PFA::HA, QPD::HA) expression levels in ΔPFA. The parasite protein SERP was used as a loading control. I) Immunofluorescence verifies export of episomally expressed PFA::HA and QPD::HA fusions to the host cell. (TIF) [file ppat.1009969.s006.tif]

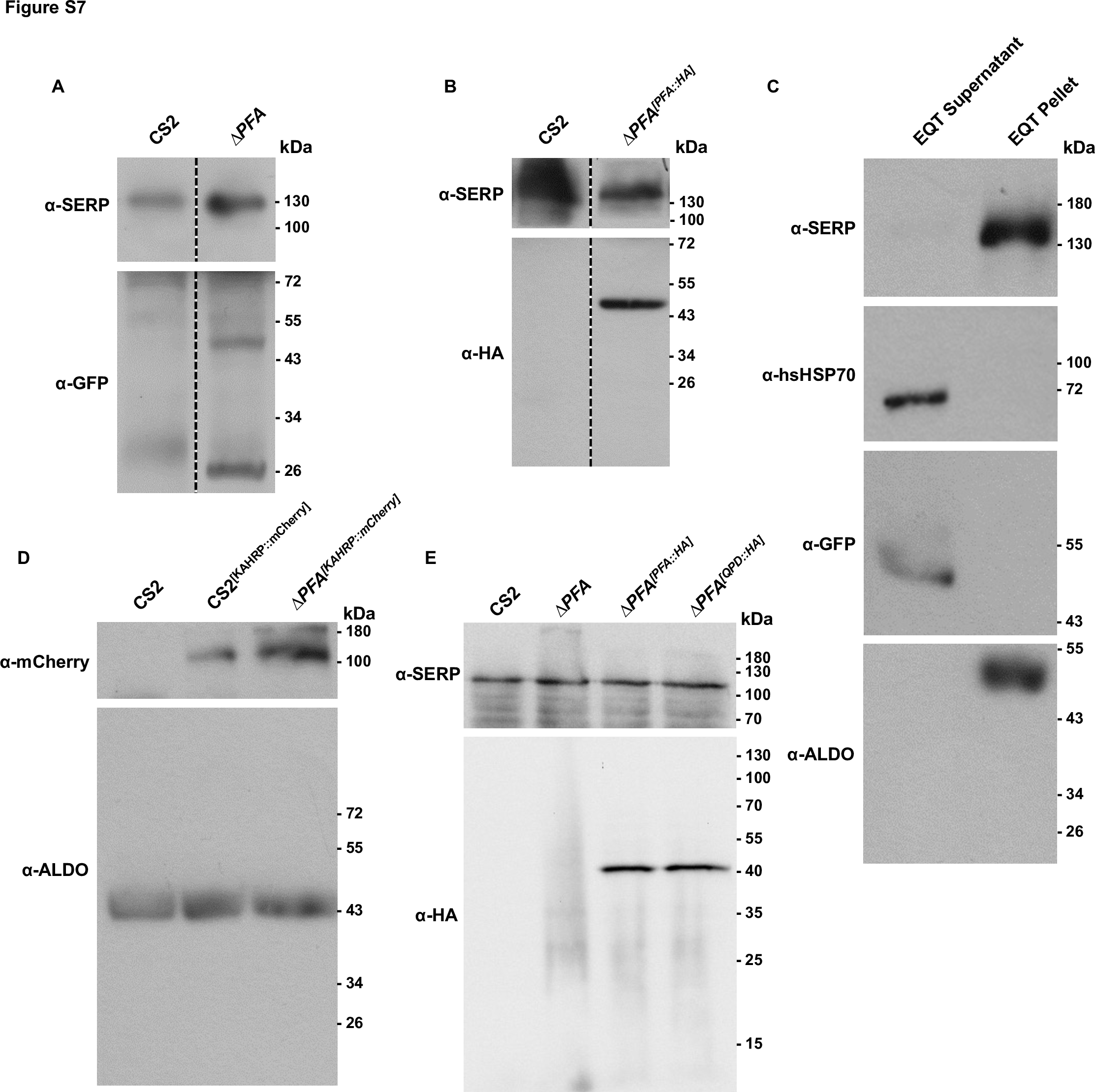

Supplement: S7 Fig — A) Western blot verifies truncation of PFA0660w in ΔPFA [Fig 1]. B) Verification of the complementation cell line ΔPFA[PFA::HA] [Fig 1D]. C) Equinatoxin experiment demonstrates export of the dPFA::GFP fusion protein to the iRBC [S1A Fig]. D) Verification of KAHRP::mCherry expression in CS2[KAHRP::mCherry] and ΔPFA[KAHRP::mCherry] [S5C Fig]. E) Comparison of episomally expressed trans-gene (PFA::HA, QPD::HA) expression levels in ΔPFA [S6H Fig]. (TIF) [file ppat.1009969.s007.tif]
