# Supplementary material for: Co-chaperone involvement in knob biogenesis implicates host-derived chaperones in malaria virulence
Source: PLoS Pathog. 2021 Oct 6;17(10):e1009969. doi: 10.1371/journal.ppat.1009969 (PMC8544838; doi:10.1371/journal.ppat.1009969)
Supplement: Attachment — Submitted filename: R2_Response_Diehl_24_8.docx [file ppat.1009969.s017.docx]

**Reviewer #1 (Evidence, reproducibility and clarity (Required)):**

**Review of "Co-chaperone involvement in knob biogenesis implicates host-derived chaperones in malaria virulence." by Diehl et al for PLOS Pathogens.**

****Major Comments.****

**1. In this paper the function of Plasmodium falciparum exported protein PFA66, is investigated by replacing its functionally important dnaJ region with GFP. These modified parasites grew fine but produced elongated knob-like structures, called mentulae, at the surface of the parasites infected RBCs. Knobs are elevated platforms formed by exported parasite proteins at the surface of the infected RBC that are used to display PfEMP1 cytoadherance proteins which help the parasites avoid host immunity. The mentulae still display some PfEMP1 and contain exported proteins such as KAHRP but can no longer facilitate cytoadherence. Complementation of the truncated PFA66 with full length protein restored normal knob morphology however complementation with a non-functional HPD to QPD mutant did not restore normal morphology implying interaction of the PFA66 with a HSP70 possibly of host origin is important for function. While a circumstantial case is made for PFA66 interacting with human HSP70 rather than parasite HSP70-x, is there any direct evidence for this eg, protein binding evidence? I feel that without some additional evidence for a direct interaction between PFA66 and human HSP70 then the paper's title is a little misleading.**

>We thank the reviewer for their kind words. We have spent considerable time generating data on stimulation of human HSP70s by the J-domain of PFA66, which is now presented in Figure 6. In the revised manuscript we explain the need to distinguish between chaperone-cochaperone and chaperone-substrate interaction when carrying out functional assays, and resolve this issue by uncoupling these distinct mechanisms. Under these conditions, the J-domain of PFA66 can stimulate HsHSP70, HsHSC70 and PfHSP70-X. As explained in the revised manuscript, it is likely that earlier studies could not distinguish between a chaperone-cochaperone interaction, and a chaperone-substrate interaction (lines 314-317 and 455-459) because the functional interaction between a HSP70 and its J-domain protein requires two signals, one from the J-domain and one from the substrate. If PFA66 is not a substrate for (lines 470-474) HSPA1A (*Hs*HSP70), the J-domain alone does only weakly stimulate the hydrolysis of ATP by HSP70 (Karzai and McMacken 1996). We believe that, given the nature of the experimental system (lack of a robust system for generating mutant erythrocytes), our two-pronged approach of parasite cell biology paired with biochemistry is the closest to “direct evidence” possible at this present date.

**2. Was CSA binding restored upon complementation of ∆PFA with the full-size copy of PFA66?**

>As this project grew organically and was driven by the results already obtained, we decided to use knob morphology via SEM as a “proof-of-principle” to show that we could reverse the phenotype. Thus, while we cannot comment on whether ALL functions of PFA66 are complemented, we suspect that if the knobs revert to their WT morphology, this is likely to be true for the other tested phenotypes. We do not feel that revisiting all of our assays (which would basically entail repeating almost every experiment so far carried out) would really be much more informative. We have added a note in the discussion stating (lines 470-474) “*We wish to note that we cannot unequivocally state that our complementation construct allows reversion of all the aberrant phenotypes herein investigated, however we feel it likely that all abnormal phenotypes are linked and thus our “proof-of-principle” investigation of knob/eKnob phenotypes is likely to be reflected in other facets of host cell modification and can thus be seen as a proxy for such.*”

****Minor Comments****

**3. Line 36, NPP should be NPPs if referring to the plural.**

>Changed

**4. Line 37, MC should be MCs if referring to the plural. By the way this acronym is never used in the text, it's always written 'Maurer's clefts'.**

>Changed

**5. Abstract, Line 52-53, could be changed to "uncover a new KAHRP-independent..." as it currently implies (albeit weakly) that that this is the first observation of a KAHRP-independent mechanism for correct knob biogenesis. Maier et al 2008, have previously shown that knock out of PF3D7_1039100 (J-domain exported protein), greatly reduced knob size and knock out of PHISTb protein PF3D7_0424600, resulted in knobless parasites.**

>Correct. In line with the suggestions of another reviewer, this section has been changed.

**6. In the Abstract it is mentioned that "Our observations open up exciting new avenues for the development of new anti-malarials." This is never really expanded upon in the rest of the paper and so seems like a bit of a throwaway line and could be left out.**

>Good point, changed

**7. Line 59, WHO world malaria report should be cited here since these numbers are from the report not a paper from 2002.**

>Done

**8. Line 67, Marti et al 2004 should be cited here as its published at the same time as Hiller et al 2004.**

>Our mistake. Done

**9. Line 76, I suggest using either 'erythrocyte' or 'red blood cell' throughout the text not both**.

>We now use red blood cells (RBCs) throughout

**10. Line 80, Maier et al 2008 should be referenced here**.

>Done

**11. Line 87, the authors should cite Birnbaum et al 2017 for the technique used. This is cited immediately after (line 98) in the results section but could be addressed at both points in the text.**

>Done

**12. Line 123, IFAs and live cell imaging failed to detect the PFA-GFP protein and the author proposes this is due to low expression levels. However, PFA66 is expressed at ~350 FPKM in the ring stage and previous studies from your own group have visualised it using GFP before. Is there another explanation for this such as disruption of the locus here has served to greatly reduce the expression level of the fusion protein?**

>We thank the reviewer for their insight. We have now carried out an analysis of protein abundance levels, and show that disruption of the locus does in fact lead to a lower amount of the truncated protein when compared to a GFP tagged endogenous version (Figure 1C). We feel that this analysis further supports our hypothesis, as there is even less truncated protein than we originally thought.

As far as our failure to detect the truncated protein in IFA, we wish to note that (as well as the lower abundance revealed above) the truncated protein is now distributed throughout the whole erythrocyte cytosol, not concentrated into J-dots, likely making detection difficult. Our original GFP tagged PFA66 lines (Külzer et al, 2010) did not really show a strong signal in comparison to other lines we are used to analysing. Nevertheless, we believe that our sub-cellular fractionation data (Figure S1B) clearly demonstrates the erythrocyte cytosolic localization of the truncated PFA66.

**13. Line 147, for consistency it would be best to introduce infected red blood cell (iRBC) at the beginning of the main text and use throughout the text instead of switching between 'infected human erythrocyte' and iRBC.**

>We agree, and have changed accordingly

**14. Line 153, Fig S2A does not exist.**

>We apologise, this has been changed

**15. Lines 156-158: Different knob morphologies are described with repeated reference to Fig2 and FigS2. Since multiple whole-cell SEM images are displayed in these figures it would be worth adding lettering and/or zoomed-in regions of interest highlighting examples of each aberrant knob type.**

>This has now been added to Figure S2.

**16. Line 178-179, "Although not highly abundant in either sample, the morphology of Maurer's clefts appeared comparable in both samples (data not shown)." Why is the data not shown? Representative images of Maurer's clefts from each line should be included in the supplementary figures or this in-text statement should more clearly justified.**

>Figure S3 has been adjusted to also show Maurer´s clefts in more detail. An Excel table of Data can be provided if necessary.

**17. Line 196, indirect immunofluorescence assay (IFA).**

>Changed

**18. Line 201, how was the 'non-significant difference' measured? PHISTc looks quite different by eye. Rephrase the term "significant difference" as localisation of these exported proteins was compared visually rather than quantified. Otherwise, a measure of mean fluorescence intensity could be taken for each protein as a basic comparison between the two lines. In the Figure legend of S4, the term "no drastic difference", is used suggesting this was not quantified. By the way, PHISTc appears different by the represented figure.**

>We apologise for our use of a specific term for non-statistically verified observations. The PHISTc image the reviewer comments on, was presented incorrectly (too much brightness introduced during processing) and is now correct. We mean to say that we could not (in a blinded check), tell the difference between WT and KO IFA images. Only KAHRP (in our opinion) demonstrated a different fluorescence pattern. As KAHRP has previously been implicated in knob formation, we then analysed this phenotype in more detail. A detailed analysis of the fluorescence pattern in the other IFAs does, in our eyes, not add to the story or add any real value to our observations.

**19. Line 213, you now have 3 versions for the word wild type, 'wild type', 'wild-type' and 'WT', best to choose one for consistency.**

>Changed

**20. Line 232, 'tubelike' to 'tube-like'.**

>Changed

**21. Line 279, just use 'IFA', the acronym has already been explained earlier in the text.**

>Changed

**22. Line 319, 'permeation' should be 'permeability'.**

>Changed

**23. Line 353, 'The action of host actin is known' to 'Host actin is known'.**

>Changed

**24. Line 373, 'through their role as regulators'.**

>Changed

**25. Line 402, either use 'HSP70-x' or 'HSP70-X' throughout the text.**

>Changed

**26. Line 540, the speed used to pellet the samples for sorbitol lysis assay, 1600g is quite high and could reflect RBC fragility rather than direct sorbitol induced lysis. The parasitemia is also very low, and previous published methods have used ~90% parasitemia rather than the 2% used here. We are not saying the method is wrong but please check it is accurate.**

>We used the method of our former colleague Stefan Baumeister (University of Marburg), who is an expert in analysis of NPP, thus we are sure the method is correct. We are in fact tempted to remove the NPP data as they deflect from the main narrative of the manuscript, this being the reason we include them only as supplementary data

**27. Line 479, 10µm should be 10 µM.**

>Changed

**28. In Fig 1A, the primers A, B, C etc are not explained anywhere that I can see.**

>This information has now been included in the 1A Figure legend and Table 2A.

**29. Figure 1B, I do not see any clear band for the 3' integration indicated with the *. Can a better image be shown?**

>We apologise for this. We have repeated all the integration PCRs with necessary controls and this figure has been replaced. We also include full-length gel pictures as Figure S1A.

**30. It seems from Fig 3G,H,I that the KAHRP puncta are bigger in ∆PFA but are as abundant as CS2. Given that KAHRP is associated with knobs how do you reconcile this with there being fewer knobs per unit area in ∆PFA compared to CS2 as in Fig 2B? The numbers of knobs/KAHRP spots/Objects per um2 seems to vary between Fig 2 and 3. Please provide some commentary about this.**

>We are not sure if all KAHRP spots actually label eKnobs, and it is possible that there are KAHRP “foci” that are not associated with eKnobs. We also wish to note that the data in figure 2 and 3 were produced using very different techniques. Sample preparation may lead to membrane shrinkage or stretching, and the different microscopy techniques have very different levels of resolution. For this reason we do not believe that the data from these very different independent experiments can be compared, however a comparison within a data set is possible and good practice.

**31. In the bottom panels of Fig 4, KAHRP::mCherry appears to extend beyond the glycocalyx beyond the cell. Is this an artifact?**

>We checked assembly of the figure and are sure that this was not introduced during production of the figure. Our only explanation is that WGA does not directly stain the erythrocyte membrane, but the glycocalyx. A closer examination of the WGA signal reveals that it is weaker at this point (and also in the eKnobs i, ii) so potentially the KAHRP signal is beneath the erythrocyte plasma membrane, but the membrane cannot be visualised at this point.

**32. Line 837, does this refer to 10 technical replicates or was the experiment repeated on 10 independent occasions? This should at least be done in 2 biological replicates given the range in technical replicates on the graph. Was CS2 considered as '100% lysis' or the water control described in the method? Please provide more detail.**

>This figure is the result of 10 biological and 4 technical replicates. A number of data points were removed as lying outside normal distribution (Gubbs test). The highest value within a biological replicate was set to 100% to allow comparison of results. This has now been corrected in the text.

**Reviewer #1 (Significance (Required)):**

**This is a reasonably significant publication as it describes knob defects that to my knowledge have never been observed before. Importantly, the deletion of the J domain from PFA66 is genetically complemented to restore function really confirming a role for this protein in knob development. Amino acids critical for the function of the J-domain are also resolved. Apart from some minor technical and wording issues the paper is really nice work apart from one area which is the proposed partnership of PFA66 with human HSP70 for which there is not much direct evidence. If this evidence can be provided, we think this work could be published in a high impact journal. Without the evidence, it could find a home in a mid-level journal with some tempering of the claims of PFA66's interaction with human HSP70.**

>We thank the reviewer for their support. We are sure that they will appreciate the new data we now include showing stimulation of human HSP70 by the J-domain of PFA66 (Figure 6), and I think you will agree that this significantly strengthens our manuscript.

****Referee Cross-commenting****

**There seems to be a high degree of similarity in the reviewers' comments and I think as many issues as possible should be addressed. I definitely agree that the term mentula should be not be used.**

>We have now adopted the suggestion of Reviewer 3, and use the term eKnobs.

**Reviewer #2 (Evidence, reproducibility and clarity (Required)):**

***Plasmodium falciparum* exports several proteins that contain J-domains and are hypothesized to act as co-chaperones to support partner HSP70s chaperones in the host erythrocyte, but the function of these co-chaperones is largely unknown. Here the authors provide a functional analysis of one of these exported HSP40 proteins known as PFA66 by using the selection-linked integration approach to generate a truncation mutant lacking the C-terminal substrate binding domain. While there is no fitness cost during in vitro culture, light and electron microscopy analysis of this mutant reveals defects in knob formation that produces a novel, extended knob morphology and ablates Var2CSA-mediated cytoadherence. These knob formation defects are distinct from previous mutants and this unique phenotype is exploited by the authors to show that the HSP70-stimulating "HPD" motif of PFA66 impacts rescue of the altered knob phenotype. In other HSP40 co-chaperones, this motif is critical to stimulate partner HSP70 activity, suggesting that PFA66 acts as a bona fide co-chaperone. Importantly, previous work by the Przyborski lab and others has shown that deletion PfHSP70x, the only HSP70 exported by the parasite, does not phenocopy the PFA66 mutant, implying that the partner HSP70 is of host origin. The results are exciting but I have some concerns about controls needed to properly interpret the functional complementation experiments. My specific comments are below.**

>We have endeavoured to fulfil the reviewer’s wishes, and have carried out all the requested controls (see below for details).

****Major comments****

**- The failure of the HPD mutant PFA66 to rescue the knob-defect is very interesting. However, the authors need to determine that the HPA mutant is expressed at the same level as the WT (by quantification against the loading controls in the western blots in Fig 1D and Fig S6H) and is properly exported (by IFA and/or WB on fractionated iRBCs, as done for the GFP-fused truncation in Fig S1A). Otherwise, the failure to rescue is hard to interpret. If these controls were in place, the conclusion that a host HSP70 is likely being hijacked by PFA66 is appropriate. This genetic data would be greatly strengthened by in vitro experiments with recombinant protein showing activation of a host HSP70 by PFA66, but I realize this may be out of the scope of the present study. Along these lines, it might be worth discussing the finding by Daniyan et al 2016 that recombinant PFA66 was found to bind human HSPA1A with similar affinity to PfHSP70x but did not substantially stimulate its ATPase activity, suggesting this is not the relevant host HSP70. This study is cited but the details are not discussed.**

>1. We now show, by direct comparison with a loading control, that the abundance of both fusions is at approximately the same level.

>2. We have localised both WT and HPD mutant by IFA, and verify that they are both exported normally, and the localisation is characteristic of J-dots (which would be correct, Figure S6I). We did not observe any mislocalisation due to the mutation. This is consistent with our previous data showing that J-dot localisation of exported HSP40s is dependent on the substrate binding domain (Petersen et al, 2016, IJP).

>3. We now include data showing functional interaction between the J-domain of PFA66, *Hs*HSP70, *Hs*HSC70 and *Pf*HSP70-X. As explained in the revised manuscript, it is likely that earlier studies could not distinguish between a chaperone-cochaperone interaction, and a chaperone-substrate interaction (lines 314-317 and 455-459) because the functional interaction between a HSP70 and its J-domain protein requires two signals, one from the J-domain and one from the substrate. If PFA66 is not a substrate for (lines 470-474) HSPA1A (*Hs*HSP70), the J-domain alone does only weakly stimulate the hydrolysis of ATP by HSP70 (Karzai and McMacken 1996) and could very well explain the results obtained by Daniyan et al. 2016, since their experiments were performed in the absence of any substrates other than PFA66. By fusing a known human HSP70 substrate to the PFA66 J-domain, we simplified the system, included a substrate and show that our experimental approach (explained in lines) allows the determination of the chaperone-cochaperone dynamics, revealing that the PFA66 J-domain is able to functionally interact with two major human HSP70s in the RBC in addition to *Pf*HSP70-X.

**- The authors claim that truncation of PFA66 alters the localization of KAHRP but not the other exported proteins they evaluated by IFA (Fig S4). This seems baseless as they don't apply the same imageJ evaluation to these other proteins. Similarly, the statement that KAHRP structures "appear by eye to have a lower circularity, although we were not able to substantiate this with image analysis" is subjective/qualitative and should probably be removed.**

>We mean to say that we could not (in a blinded check), tell the difference between WT and KO IFA images. Only KAHRP (in our opinion) demonstrated a different fluorescence pattern. As KAHRP has previously been implicated in knob formation, we then analysed this phenotype in more detail. A detailed analysis of the fluorescence pattern in the other IFAs does, in our eyes, not add to the story or add any real value to our observations.

The statement on the circularity has been removed according to the reviewers wishes.

**-The section title "Chelation of membrane cholesterol...causes reversion of the mutant phenotype in ∆PFA" seems an overstatement given the MBCD effect on the knob morphology is fairly weak and remains significantly abnormal.**

>The title of this section was misleading, we agree. We have retitled it “*Chelation of membrane cholesterol but not actin depolymerisation or glycocalyx degradation causes partial reversion of the mutant phenotype in ∆PFA”* to clarify that the reversion was only partial (as explained by the following text in the manuscript).

****Minor comments****

**- The DNA agarose gel image in Fig 1B is not very convincing. Most of the bands are faint and there is a lot of background/smear signal in the lanes. Also, it would help for clarity if the primer pairs used for each reaction were stated as shown in the diagram (rather than simply "WT", "5' Int" and "3' Int").**

>We apologise for this. We have repeated all the integration PCRs with necessary controls and this figure has been replaced. We also include full-length gel pictures as Figure S1A. The Figure has also been changed accordingly.

**- Given the vulgar connotation of "mentula", the authors might consider an alternative term.**

>We have now adopted the term “eKnobs” suggested by Reviewer 3.

**- lines 67-69: The authors may wish to cite a more recent review that takes into account updated Plasmepsin 5 substrate predication from Boddey et al 2013 (PMID: 23387285). For example, Boddey and Cowman 2013 (PMID: 23808341) or de Koning-Ward et al 2016 (PMID: 27374802).**

>A fair point, we have now added Koning-Ward.

**- lines 77-79: "deleted" is repetitive in this sentence.**

>Changed

**- line 115: It might be clearer to state "endogenous PFA66 promoter**"

>Changed

**- lines 131-132: "...these data suggests that deletion of the SBD of PFA66 leads to a non-functional protein." Behl et al 2019 (PMID: 30804381) showed the recombinant C-terminal region of PFA66 (residues 219-386, including the SBD truncated in the present study) binds cholesterol. The authors may wish to mention this along with their reference to Kulzer et al 2010 showing PFA66 segregates with the membrane fraction, suggesting cholesterol is involved in J-dot targeting.**

>We should have noted this connection and thank the reviewer for bringing it to our attention. This section has been revised to include this important information.

**- line 198: It's not clear what is meant by "+ve" here and afterward. Please define.**

>We have now changed this to “structures labelled by anti-KAHRP antibodies”, or merely “KAHRP”.

**- lines 749-750: "Production of PFA and NEO as separate proteins is ensured with a SKIP peptide". Translation of the 2A peptide does not always cause a skip (see PMID: 24160265) and often yields only about 50% skipped product (for example, PMID: 31164473). Because of the close cropping in the western blots in Fig 1C or S1A this is difficult to assess. Is a larger unskipped product also visible? Beyond this one point, it is general preferable that the blots not be cropped so close.**

>A very valid point. We have repeated the experiment and do detect a non-skipped portion. The figure has been changed accordingly and we have added a note in the text (Lines 109-110). We do not believe that the presence of a non-skipped fusion invalidates our results, especially considering the low abundance of both skipped and non-skipped variants (new Figure 1C). The domain added in the non-skipped version of the protein (Neo^R^) apparently is not able to serve as substrate binding domain for the PFA66 truncation construct.

**- lines 867-868: Explain more clearly what "Cy3-caused fluorescence" is measuring.**

>The Cy3 channel refers to anti-var2CSA staining, and we have now included this information.

**- Several figure legends would benefit from a title sentence describing what the figure is about (ie, Fig legends 1, 3, 5, S1, S5 & S6)**

>This has been added.

**Reviewer #2 (Significance (Required)):**

**This manuscript by Diehl et al reports on the function of the exported P. falciparum J-domain protein PFA66 in remodeling the infected RBC. Obligate intracellular malaria parasites export effector proteins to subvert the host erythrocyte for their survival. This process results in major renovations to the erythrocyte, including alteration of the host cell cytoskeleton and formation of raised protuberances on the host membrane known as knobs. Knobs serve as platforms for presentation of the variant surface antigen PfEMP1, enabling cytoadherence of the infected RBC to the host vascular endothelium. This process is of great interest as it is critical for parasite survival and severe disease during in vivo infection. The basis for trafficking of exported effectors within the erythrocyte after they are translocated across the vacuolar membrane is not well understood but is known to involve chaperones. This is a particularly interesting study in that it provides evidence in support of the hypothesis, initially proposed nearly 20 years ago, that the parasite hijacks host chaperones to remodel the erythrocyte. This is biologically intriguing and also suggests new therapeutic strategies targeting host factors that would not be subjected to escape mutations in the parasite genome. The work will be of interest to the those studying exported protein trafficking and/or virulence in Plasmodium (such as this reviewer) as well as the broader chaperone and host-pathogen interaction fields.**

****Referee Cross-commenting****

**I also agree with similarity in comments. Some additional discussion on the failure to localize the PFA66 truncation by live FL is warranted, as noted by reviewer #1. Seems likely that either the level of PFA66 protein is reduced by the truncation or the truncated PFA66 is dispersed from J-dots and harder to visual when diffuse instead of punctate. In either case, the complementing copy (WT or QPD) should be visualized by IFA.**

>We are happy to report that this data is now included (Figure S6I), and we also suspect that our failure to detect the truncation is due to a reduced (compared to GFP-tagged full-length) protein abundance. As we state in our answer to reviewer 1:

“We have now carried out an analysis of protein abundance levels, and show that disruption of the locus does in fact lead to a lower amount of the truncated protein when compared to a GFP tagged endogenous version (Figure 1C). We feel that this analysis further supports our hypothesis, as there is even less truncated protein than we originally thought. As far as our failure to detect the truncated protein in IFA, we wish to note that (as well as the lower abundance revealed above) the truncated protein is now distributed throughout the whole erythrocyte cytosol, not concentrated into J-dots, likely making detection difficult. Our original GFP tagged PFA66 lines (Külzer et al, 2010) did not really show a strong signal in comparison to other lines we are used to analysing. We believe that our sub-cellular fractionation data (Figure S1B) clearly demonstrates the erythrocyte cytosolic localization of the truncated PFA66”.

**Reviewer #3 (Evidence, reproducibility and clarity (Required)):**

**The data are for the most part well controlled and reveal a potential function for PFA66 in knob formation. The assays are state of the art and the data provides insight into knob formation.**

**However, some conclusions are not fully supported by the data. For example, 'uncover a KAHRP-independent mechanism for correct knob biogenesis' (line 52-53) is not supported by the data because PFA66 truncation could result in misfolding of KAHRP and thus lead to knob biogenesis defects.**

>We meant to imply that not only perturbations/absence of KAHRP lead to aberrant knobs. This is now changed to “…uncover a new KAHRP-independent molecular factor required for correct knob biogenesis.”.

**The other major issue is that despite having a complemented parasite line in hand, the parental parasite line is used as a control for almost all assays. This is a critical issue because an alternative explanation for their data would be that expression of truncated PFA66 leads to expression of a misfolded protein that aggregates in the host RBC OR it clogs up the export pathway and indirectly leads to knob biogenesis defects. It is surprising that the authors do not test the localization of dPFA using microscopy especially since it is tagged with GFP. While the complemented parasite line does revert back, this could also be due to the fact that the complement overexpresses the chaperone helping mitigate issues caused by the truncated protein.**

>1)As all virulence characteristics we monitor in this study have been verified many times in the parental CS2 parasites in the literature, we think that the best comparative control is indeed the truncated cell line. The large part of our study aimed to characterize differences in various characteristics upon inactivation of PFA66 function, and for this reason we used the parental WT line as a control.

>2) We now show correct localisation of both WT and HPD mutants, and can show that they are correctly exported.

>3) Our failure to localise the PFA truncation is likely due to a reduced abundance which we now show via immunoblotting (Figure 1C, and answer to reviewers 1 and 2). Our subcellular fractionation verifies that all truncated protein is soluble and present in the infected erythrocyte, thus blockage of any PV export pathway or protein aggregation can be excluded and cannot explain the phenotype we observe. Other proteins were still normally exported (Figure S4), further supporting a functional export pathway. Indeed, as reported by ourselves and our colleagues (particularly from the Spielmann laboratory, Mesen-Ramirez et al 2016, Grüring et al 2012), blockage of the export pathway is likely to lead to non-viable parasites as the PTEX translocon seems to be the bottleneck for export of a number of proteins, many of which are essential for parasite survival.

**Reviewer #3 (Significance (Required)):**

**The malaria-causing parasite extensively modifies the host red blood cell to convert the host into a suitable habitat for growth as well as to evade the immune response. It does so by exporting several hundred proteins into the host cell. The functions of these proteins remain mostly unknown. One parasite-driven modification, essential for immune evasion, is the assembly of 'knob' like structures on the RBC surface that display the variant antigen PfEMP1. How these knobs are assembled and regulated is unknown.**

**In the current manuscript, Diehl et al target an exported parasite chaperone from the Hsp40 family, termed PFA66. The phenotypic observations described in the manuscript are quite spectacular and well characterized. The truncation of PFA66 results in some abnormal knob formation where the knobs are no longer well-spaced and uniform but instead sometimes form tubular structures termed mentulae. The mechanistic underpinnings driving the formation of mentulae remain to be understood but that will probably several more manuscripts to be deciphered.**

>We thank the reviewer for their kind comments, and also for the recognition that this current manuscript is merely the exciting beginning of a story!

****Major Comments:****

>General comment on the use of controls: The large part of our study aimed to characterize differences in various characteristics upon inactivation of PFA66 function, and for this reason we used the parental WT line as a control. Using the complementation line as a control in this context would not truly reflect the effect of PFA66 truncation, as PFA66::HA was not expressed from an endogenous locus, but rather from an episomal plasmid. This itself may result in expression levels which differ from WT, and thus this parasite line cannot be seen as the gold-standard control for assaying PFA66 function. Our complementation experiments were initially designed to verify that phenotypic changes ONLY related to inactivation of PFA66 function and were (as unlikely as this is) not due to second site changes during the genetic manipulation process. To avoid lengthy and not really very informative analysis of the complementation line, we used knob morphology via SEM as a “proof-of-principle”. However, as the reviewer is formally correct, we have added a passage to the discussion stating that “*We wish to note that we cannot unequivocally state that our complementation construct caused reversion of all the aberrant phenotypes herein investigated, however we feel it likely that all abnormal phenotypes are linked and thus our “proof of principle” investigation of knob/eKnob phenotypes is likely to be reflected in other facets of host cell modification and can thus be seen as a proxy for such.*“.

**Fig 3: The control used here is the parental line. Was there a reason why the complemented parasite line was not used as the control? Showing that the KAHRP localization and distribution is restored upon complementation would greatly increase the confidence in the phenotype.**

>Please see our general comments above.

**Fig 5: The data showing a defect in CSA binding are convincing but again only the parental control is used and not the complemented parasite line. The complemented parasite line should be used as a control for the PFA binding mutant.**

>Please see our general comments above, and also our response to reviewer 1.

**In 5D, the defect in dPFA seems to be occur to a lesser degree than Fig. 2C. How many biological replicates are shown in each of these figures? The figure legend says 20 cells were quantified via IFA but were these cells from one experiment? The expression of mentulae seems quite variable, while the authors mention '22%' (line 164), it seems in most other experiments, its more ~10% (5D and S6B, D-E). Were these experiments blinded?**

>As the reviewer is likely aware, subtle differences in parasite culture conditions, stage, fixation, SEM conditions and length of time in culture between time experimental time points can lead to variations in results. Due to the time required to generate the data for figure 5, these experiments took place months after the original (i.e. Figure 2C) analysis. It is not possible to directly compare the results of these two independent experiments, however it is possible to compare the results of the parasite lines included within each set of experimental data.

**Fig S6G: The staining suggests that most PfEMP1 in is not exported, in any parasite line. Staining for PfEMP1 is technically challenging and these data are not enough to show that expression level is 'similar' (Line 279-280). It may be more feasible to use the anti-ATS antibody and stain for the non-variant part of PfEMP1 (Maier et al 2008, Cell).**

>It is well known that a large portion of PfEMP1 remains intracellular. This figure does not aim to differentiate between surface exposed and internal PfEMP1, but merely to show that similar TOTAL PfEMP1 is expressed in the deletion line, and also that the parasites have not undergone a switching event away from *var2CSA* which would lead to loss of CSA binding ability.

**Lines 320-322: The logic of why increased robustness of the RBC membrane would lead to faster parasite growth is confusing. It is likely that the loss of PfEMP1 expression leads to faster growth. The loss of NPP is minimal and may not cause growth defects in rich media.**

>As far as we can detect, there is no loss of total PfEMP1 expression (as verified by figure S6G), but rather a drop in surface exposure and functionality, which is unlikely to affect parasite growth rates. What we intended to say was that the NPP assay is influenced by fragility of the erythrocyte, and therefore a stiffer erythrocyte may be more resistant to sorbitol-induced lysis. As the NPP result does not really add much to the main narrative of this manuscript, we would prefer not to invest unnecessary effort for a minimal potential readout. Indeed, we are tempted to remove the NPP data as they deflect from the main findings of the manuscript, this being the reason we include them only as supplementary data

**Lines 433-434: These data do support a function for HsHsp70 but these data are among many others that have previously provided circumstantial evidence for its role in host RBC modification. May be a co-IP would help support these conclusions better.**

>Despite all our best efforts and publications, we have been unable to detect this interaction in co-IP or crosslink experiments, although we were successful in detecting interactions between another HSP40 (PFE55) and *Hs*HSP70 (Zhang et al, 2017). Although this is disappointing, it may be explained due to the transient nature of HSP40/HSP70 interactions, and also the fact that detection of JDP-HSP70 interactions by co-IP only works if HSP70 recognizes the JDP as substrate. As we now show (new data, Figure 6), this issue can be circumvented (in functional assays) by use of a model substrate fused to the J-domain of PFA66. We agree that our suggestion (that parasite HSP40s functionally interact with human HSP70) is not novel (we and others have noted this possibility for over 10 years), however the challenging nature of the experimental system makes it very difficult to show direct evidence of the importance of this interaction *in cellula*. Over the past decade we have use numerous experimental approaches to try to address this but have always been confounded by technical challenges. In 2017 the corresponding author took a sabbatical to attempt manipulation of hemopoietic stem cells to reduce HSP70 levels in erythrocytes, however it appears (unsurprisingly) that *Hs*HSP70 is required for stem cell differentiation, and thus this tactic was not followed further. The authors believe that, due to the lack of the necessary technology, indirect evidence for this important interaction is all that can realistically be achieved at this time, and this current study is the first to provide such evidence. We would further like to note that a successful co-IP would not directly verify a functional interaction between PFA66 and *Hs*HSP70, but could also reflect a chaperone-substrate interaction between these proteins, and is therefore not necessarily informative.

On the positive side, our manuscript now includes data showing that the J-domain from PFA66 can stimulate the ATPase activity of both human and parasite derived Hsp70 (see lines 305-332 and our reply to reviewers 1 and 2). We believe that this robust data validates a potential functional interaction between PFA and human chaperones, and also distinguishes between a chaperone-substrate and chaperone-cochaperone interaction.

****Minor Comments:****

**Fig1: The bands are hard to see in WT and 3’Int. May be a better resolution figure would help? Also, the schematic shows primers A-D but the figure legend does not refer to them. It would be useful to the reader to have the primers indicated above the PCR gel along with the expected sizes.**

> We apologise for this. We have repeated all the integration PCRs with necessary controls and this figure has been replaced. We also include full-length gel pictures as Figure S1A The Figure has also been changed accordingly.

**Fig S1: The NPP data could be improved if tested in minimal media. It has been shown that NPP defects do not show up in rich media (Pillai et al 2012, Mol. Pharm. PMID: 22949525). Does complementation restore NPP and growth rate?**

> As the NPP result does not really add much to the main narrative of this manuscript, we would prefer not to invest unnecessary effort for a minimal potential readout. Indeed, we are tempted to remove the NPP data as they deflect from the main findings of the manuscript, this being the reason we include them only as supplementary data. Likewise the complementation experiments are, we feel, unnecessary.

**Fig 4: It is not clear what the line scan analysis are supposed to show. What does ‘value’ on the y-axis mean?**

>These are line scans of fluorescence intensity (arbitrary units) along the yellow arrows shown on the fluorescent panels. This is now indicated in the figure legend.

**Fig S5D: Maybe it was a problem with the file but no actin staining is visible.**

>The actin stain was visible on the screen, but unfortunately not in the PDF. We have applied (suitable) enhancement to produce the images in the new version.

**Fig 6: A model for mentulae formation is not really proposed. Only what the authors expect the mentulae to look like.**

>We have changed the legend to reflect this “Figure 7. Proposed model for eKnob formation and structure.”. We do propose that runaway extension of an underlying spiral protein may lead to eKnobs, thus would like to keep the word “formation”.

**Lines 312-313: It is not clear what 'highly viable' means, parasites are either viable or not.**

>This has been changed.

**Lines 400-405: The authors forgot to cite a complementary paper that showed no virulence defect upon 70x knockout or knockdown (Cobb et al mSphere 2017). Those data also support a role for HsHsp70.**

>We apologise for the omission. This is now included.

****Referee Cross-commenting****

**I agree, the comments are pretty similar. The authors could tone down their conclusions or add more data to support their conclusions. May be call them elongated knobs or eKnobs, instead of mentula?**

>We think the addition of robust data on PFA stimulation of HSP70s underpins our conclusions and believe they are appropriate given all the data now included. We have removed the offending term and adopted eKnobs.
