# Supplementary material for: Co-chaperone involvement in knob biogenesis implicates host-derived chaperones in malaria virulence
Source: PLoS Pathog. 2021 Oct 6;17(10):e1009969. doi: 10.1371/journal.ppat.1009969 (PMC8544838; doi:10.1371/journal.ppat.1009969)
Supplement: Attachment — Submitted filename: Diehl_Revision_ReviewersComments.docx [file ppat.1009969.s018.docx]

*>We thank all reviewers for their helpful comments which have helped to improve this manuscript.*

**Reviewer #1: All the minor issues from the previous version appear to have been addressed.**

**Reviewer #2: Minor comments**

-Line 122: Please state the expected fusion sizes for the skipped and unskipped as well as the PFA66-GFP fusion in the text and/or in the figure. I would also recommend providing a reference to the observation that these T2A fusions typically only skip ~50% of the time in Plasmodium, such as PMID 24160265 or 31164473.

*>This information, and the reference has been added.*

-Line 911: Given that ~50% of the protein is not skipped, I would change “ensured” to something like “mediated”. Also, the * and + designations on the WB should be described in the figure or figure legend.

*>This has been adjusted*

-Figure S1B: it appears the 43 kD marker is pointing to the top of the anti-GFP blot. I assume this should be the bottom of the Aldolase blot. Please adjust.

*>This has been corrected*

-The complementation line is introduced as ∆PFA[PFA::HA] in line 132 but then this name is not used in lines 185-187 when it is introduced again as a control for the eKnob phenotype. I assume it’s the same line – if so I would refer to it consistently to avoid any confusion.

*>We apologise for this omission, and this designation is now included as suggested*

-Were specific length or width thresholds use to quantify knob morphology categories in Figure 2C, 5D and S6B,D,E or was this done by eye? I didn’t find this in the methods or legends – sorry if I missed it but if it is not there, please add clarification on how the quantification was done.

*>We have now added the following information “For purposes of quantification, we manually counted and classified by sight knobs/eKnobs to one of three classes”*

-Line 192: “the lumen of these eKnobs was often extremely dense” It appears that it is not the lumen (or core) but the tip of the eKnob that is electron dense by TEM while the lumen of the eKnobs shown in Fig 2D, 3E and S3 is generally less dense. The tomography seems to show a dense core but I’m not seeing it in the TEMs being described here.

*>Indeed there was a certain heterogeneity in the electron density, but we believe that on balance, many of the eKnobs did show an electron dense lumen (figure 2 zoom, figure S3 bottom left and bottom right). We think that our text allows for this variability by the use of the word “often” “The lumen of these eKnobs was often extremely electron dense, hinting at their molecular relation to knobs. Occasionally we observed membrane-bound structures extending from RBCs infected with CS2 parasites, but the lumen of these structures was not electron dense; therefore, these structures cannot be classed as eKnobs.”*

-Line 219-220: Were these other exported proteins quantified in the same way? The main text here states “no significant difference” which implies a statistical test was applied but the legend for Fig S4 says “no drastic difference”. From the response comments, it appears that the authors were unable to identify a difference by eye in a blinded check of the images. If so, “no significant difference” is misleading and should be removed from the text.

*>We apologise for the incorrect usage of the phrase and now say “no dramatic difference” in line with the reviewer’s suggestion.*

-Line 1005: The legend indicates GBP was detected in S1B but it is not there in the figure.

*>This appears to have been a fragment left over from a previous version of the manuscript and has now been deleted.*

-Line 1023-1025: Please state the expected size of the KAHRP-mCherry fusion in the legend.

*>This information has now been added (92kDa considering cleavage of PEXEL)*

Line 326: references #43 and #44 are not right here. I think you mean Daniyan et al (#39) and Day et al 2019 (which appears to have been inadvertently deleted from the revised manuscript).

*>This has been corrected, many thanks for picking this up.*

-Lines 364-366: The authors should note that some NPP phenotypes are obscured by rich media (for example, Gupta et al 2020, PMID: 32069335). The co-chaperone function of PFA66 could play a role in supporting the establishment of PSAC/NPPs at a level that does not produce a fitness cost without nutrient limitation. Seems this warrants a comment in the discussion.

*>A very valid point, which we now address in the discussion “Alternatively, growth in rich media may have obscured a more subtle effect on NPP, and future studies may wish to address this.”*

*Grammar*

-Line 238: should be “imaged by STED microscopy”

*>Corrected*

-Line 469: “lead to misleading” is repetitive. Consider revising to “produce misleading”

*>Changed*

-Line 951: “remaining” and “remained” is repetitive. Consider revising.

*>Changed*

**Reviewer #3:**

Lines 329-228: The JDS isn’t defined, what were the amino acid numbers?

*>This has now been added (amino acids 81-148)*

Line 655: The ‘S’ in JDS is lowercase unlike in the rest of the manuscript.

*>Corrected*

Line 674: What was the source of ULP1 protease?

*>The Ulp1 protease was produced in the Mayer laboratory, this has now been indicated.*

Line 714: There’s a typo, ‘JDH’ instead of ‘JDS’

*>This has now been changed*
